# Epigenome-wide association study of DNA methylation in panic disorder

**DOI:** 10.1186/s13148-016-0307-1

**Published:** 2017-01-21

**Authors:** Mihoko Shimada-Sugimoto, Takeshi Otowa, Taku Miyagawa, Tadashi Umekage, Yoshiya Kawamura, Miki Bundo, Kazuya Iwamoto, Mamoru Tochigi, Kiyoto Kasai, Hisanobu Kaiya, Hisashi Tanii, Yuji Okazaki, Katsushi Tokunaga, Tsukasa Sasaki

**Affiliations:** 1 Department of Human Genetics, Graduate School of Medicine, The University of Tokyo, 7-3-1 Hongo, Bunkyo Ward, Tokyo, 113-0033 Japan; 2 Graduate School of Clinical Psychology, Teikyo Heisei University Major of Professional Clinical Psychology, 2-51-4 Higashiikebukuro, Toshima Ward, Tokyo, 171-0014 Japan; 3 Department of Psychiatry and Behavioral Sciences, Tokyo Metropolitan Institute of Medical Science, 2-1-6 Kamikitazawa, Setagaya Ward, Tokyo, 156-8506 Japan; 4 Division for Environment, Health and Safety, The University of Tokyo, 7-3-1 Hongo, Bunkyo Ward, Tokyo, 113-0033 Japan; 5 Department of Psychiatry, Shonan Kamakura General Hospital, 1370-1 Okamoto, Kamakura City, Kanagawa 247-8533 Japan; 6 Department of Molecular Brain Science, Graduate School of Medical Sciences, Kumamoto University, 1-1-1 Honjo, Chuo Ward, Kumamoto City, Kumamoto 860-8556 Japan; 7 Department of Neuropsychiatry, Teikyo University School of Medicine, 2-11-1 Kaga, Itabashi Ward, Tokyo, 173-0003 Japan; 8 Department of Neuropsychiatry, Graduate School of Medicine, The University of Tokyo, 7-3-1 Hongo, Bunkyo Ward, Tokyo, 113-0033 Japan; 9 Panic Disorder Research Center, Warakukai Med Corp, 3-9-18 Akasaka, Minato Ward, Tokyo, 107-0052 Japan; 10 Department of Psychiatry, Institute of Medical Life Science, Graduate School of Medicine, Mie University, 2-174 Edobashi, Tsu City, Mie 514-8502 Japan; 11 Department of Psychiatry, Koseikai Michinoo Hospital, 1-1 Nijigaokamachi, Nagasaki City, Nagasaki 852-8055 Japan; 12 Department of Physical and Health Education, Graduate School of Education, The University of Tokyo, 7-3-1 Hongo, Bunkyo Ward, Tokyo, 113-0033 Japan

**Keywords:** DNA methylation, Panic disorder, Epigenome-wide association study, Epigenetics, Psychiatric disorder

## Abstract

**Background:**

Panic disorder (PD) is considered to be a multifactorial disorder emerging from interactions among multiple genetic and environmental factors. To date, although genetic studies reported several susceptibility genes with PD, few of them were replicated and the pathogenesis of PD remains to be clarified. Epigenetics is considered to play an important role in etiology of complex traits and diseases, and DNA methylation is one of the major forms of epigenetic modifications. In this study, we performed an epigenome-wide association study of PD using DNA methylation arrays so as to investigate the possibility that different levels of DNA methylation might be associated with PD.

**Methods:**

The DNA methylation levels of CpG sites across the genome were examined with genomic DNA samples (PD, *N* = 48, control, *N* = 48) extracted from peripheral blood. Methylation arrays were used for the analysis. *β* values, which represent the levels of DNA methylation, were normalized via an appropriate pipeline. Then, *β* values were converted to *M* values via the logit transformation for epigenome-wide association study. The relationship between each DNA methylation site and PD was assessed by linear regression analysis with adjustments for the effects of leukocyte subsets.

**Results:**

Forty CpG sites showed significant association with PD at 5% FDR correction, though the differences of the DNA methylation levels were relatively small. Most of the significant CpG sites (37/40 CpG sites) were located in or around CpG islands. Many of the significant CpG sites (27/40 CpG sites) were located upstream of genes, and all such CpG sites with the exception of two were hypomethylated in PD subjects. A pathway analysis on the genes annotated to the significant CpG sites identified several pathways, including “positive regulation of lymphocyte activation.”

**Conclusions:**

Although future studies with larger number of samples are necessary to confirm the small DNA methylation abnormalities associated with PD, there is a possibility that several CpG sites might be associated, together as a group, with PD.

**Electronic supplementary material:**

The online version of this article (doi:10.1186/s13148-016-0307-1) contains supplementary material, which is available to authorized users.

## Background

Panic disorder (PD) is a major anxiety disorder characterized by recurrent unexpected panic attacks and anticipatory anxiety. According to previous twin and family studies [1–3], PD is considered to be a multifactorial disorder emerging from the interactions between multiple genetic and environmental factors. Recently, genome-wide association studies (GWASs), whole-exome sequencing and meta- analyses were performed [4–8] and identified transmembrane protein 132D (*TMEM132D*) and catechol-O-methyltransferase (*COMT*) as PD susceptibility genes [4, 5]. However, there would be other genetic factors associated with PD.

Epigenetics is one of the biological fields that is considered to play an important role in the etiology of complex diseases [9]. The term “epigenetics” is now generally understood to refer to potentially heritable and functionally relevant to gene expression and chromatin structure with no changes to genetic sequences [9, 10]. DNA methylation is one of the major forms of epigenetic modifications that was found to play important roles in the context of gene regulation [11]. Moreover, a part of the DNA methylation is reported to be involved in the pathogenesis of psychiatric disorders, including anxiety disorders [12, 13].

Previous studies on DNA methylation in anxiety disorders have focused mainly on candidate genes that were reported to be involved in the stress response, neurotransmission, and neuroplasticity [14]. One recent study conducted in social anxiety disorder (SAD) patients reported an association between SAD and oxytocin receptor (*OXTR*) gene hypomethylation [15]. Hypomethylation of the promoter and intron 2 region in another candidate gene, glutamate-decarboxylase 1 (*GAD1*), was also reported in PD patients [16]. Another preliminary study showed that CpG sites in monoamine oxidase A (*MAOA*) were significantly less methylated in PD patients than in healthy controls [17, 18] and negative life events were associated with this lower level of DNA methylation [18]. In another study, solute carrier family 6, member 4 (*SLC6A4*) and serotonin transporter (*SERT*) were examined in children with anxiety disorders before and after cognitive behavior therapy; a DNA methylation change in *SLC6A4* was related to response to the psychological therapy, as responders had increased *SLC6A4* methylation [19]. Methylation of another neurotransmitter transporter, noradrenaline transporter (*NET*), also known as solute carrier family 6, member 2 (*SLC6A2*), was also studied in subjects with PD and hypertension; results showed that DNA hypermethylation in the promoter region of *NET* caused *NET* gene silencing through the binding of methyl-CpG binding protein 2 (MeCP2), a methylation-related inhibitory transcription factor [20, 21]. However, results from another study did not support the finding of significant changes in *SLC6A2* promoter methylation in the patients with PD or major depressive disorder [22]. Overall, the results from such previous studies suggest the importance of DNA methylation abnormalities in the pathogenesis of PD, although the number of studies and the sample sizes have been limited and most of the findings have not been confirmed in replication studies.

Recently, a genome-wide approach has enabled the examination of DNA methylation patterns without any prior information. A methylation array (Infinium® Human Methylation 450 K BeadChip, Illumina Inc., San Diego, CA, USA) can simultaneously detect the DNA methylation status of more than 480,000 cytosine residues across the genome. This array has been used to successfully identify DNA methylation marks related to aging [23, 24], leukocyte subsets [25], smoking [26], and disease outcomes [27, 28]. As far as we know, there has been no report of the examination of genome-wide DNA methylation patterns in PD.

In this study, we performed an epigenome-wide association study (EWAS) of PD using the array technology. DNA samples extracted from peripheral blood were utilized. Although an EWAS using brain tissue would be more appropriate for identifying disease-associated differentially methylated positions (DMPs), peripheral blood is more accessible and might enable the development of diagnostic biomarkers. Here, we examined the genome-wide DNA methylation profiles of 48 PD subjects and 48 age- and sex-matched control subjects to investigate aberrant differences in DNA methylation that are related to PD.

## Results

### Quality check of the DNA methylation array data

In the DNA methylation array analysis, each probe signal for a sample had a detection *P* value calculated as the probability that a target signal is distinguishable from the negative controls to show the overall probe performance. We confirmed that more than 99% of all probes in all samples had a detection *P* value ≤0.05, showing that the overall performance of the assay was high. Principal component analysis using probes on the X chromosome was performed to predict the gender of samples in this study. The result showed that all samples were correctly labeled in the gender groups (Additional file 1: Figure S1). Density plots of the *β* values were prepared from the raw data of each sample for a visual inspection. All plots showed a standard bimodal distribution of the *β* values (Additional file 1: Figure S2) with the same characteristics of the distribution described in a previous study [29]. The distribution of DNA methylation was bimodal with a minority of probes showing intermediate DNA methylation levels. The DNAm age was estimated using the results of approximately 350 probes, and the Pearson’s correlation coefficients between the estimated DNAm age and chronological age were calculated to be 0.77 and 0.90 in PD and control groups, respectively, which is considered to support the data quality of this method (Additional file 1: Figure S3).

### Prediction of the distribution of leukocyte subsets

**Fig. 1:**
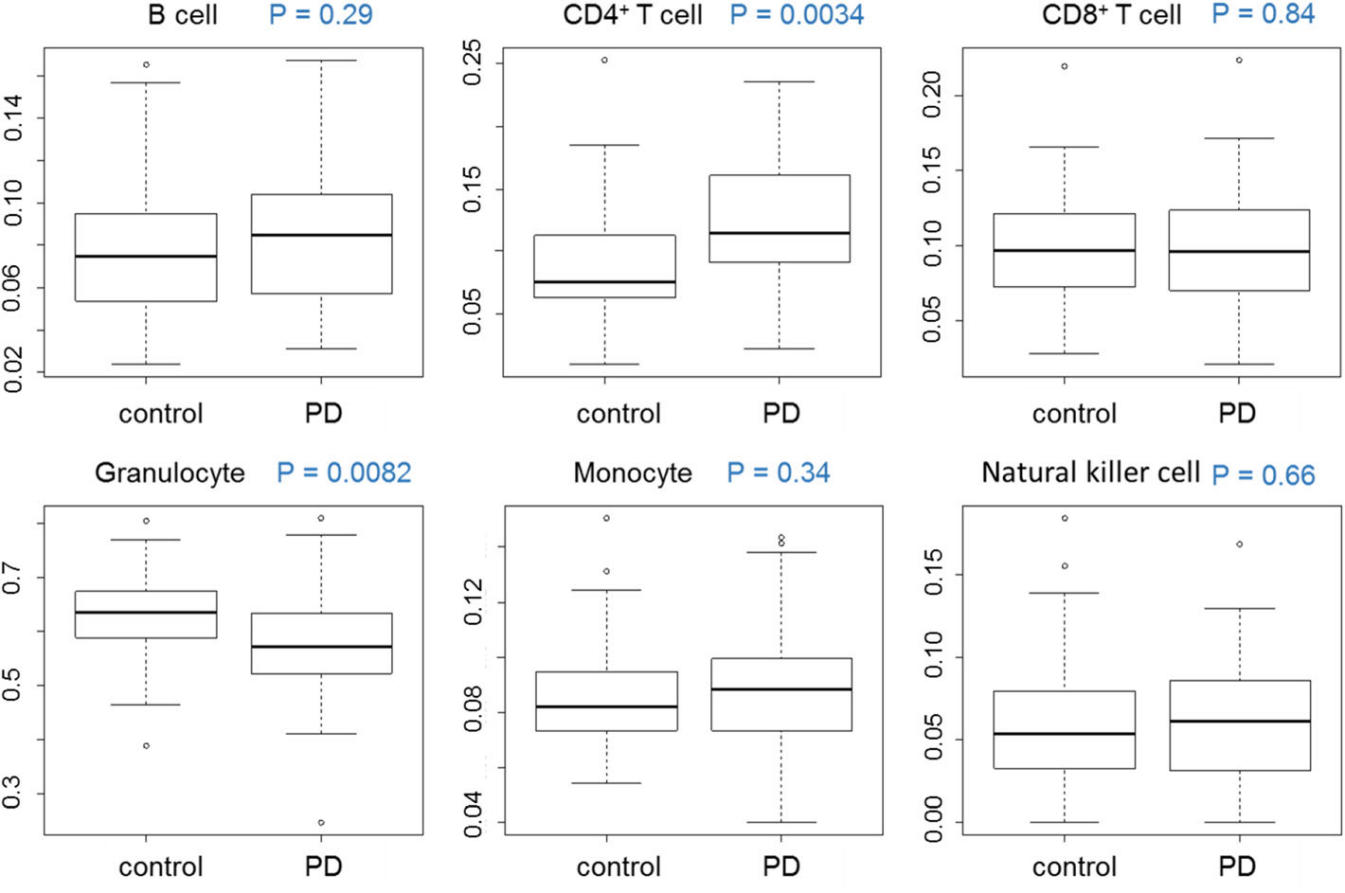
The estimated proportions of leukocyte subsets. The proportions of leukocyte subsets (natural killer cell, B cell, CD4^+^ T cell, CD8^+^ T cell, monocyte, and granulocyte) were estimated using the results of the DNA methylation array. Wilcoxon rank-sum tests using estimated proportions of leukocyte subsets were performed between the PD and control subjects. *P* values are indicated in *blue characters*. Significance level after the Bonferroni correction was set as *α* = 0.005

The proportions of leukocyte subsets (natural killer cells, B cells, CD4^+^ T cells, CD8^+^ T cells, monocytes, and granulocytes) were estimated from the DNA methylation array data using a published algorithm [30]. Wilcoxon’s rank-sum tests using the estimated proportion of each leukocyte subset were performed to examine whether the compositions of leukocyte cells differed between the PD and control subjects. The proportion of CD4^+^ T cells was significantly higher in the PD subjects than in the control subjects (*P* = 0.0034) (Fig. 1). To further interpret the result of the prediction of leukocyte subsets, the abundance measures of plasmablasts, CD8^+^CD28^−^CD45RA^−^ T cells, naive CD8^+^T cells, and naive CD4^+^ T cells were estimated and compared between PD and control subjects, resulting that no significant difference was observed (Additional file 1: Figure S4).

### EWAS of PD and control subjects

**Fig. 2:**
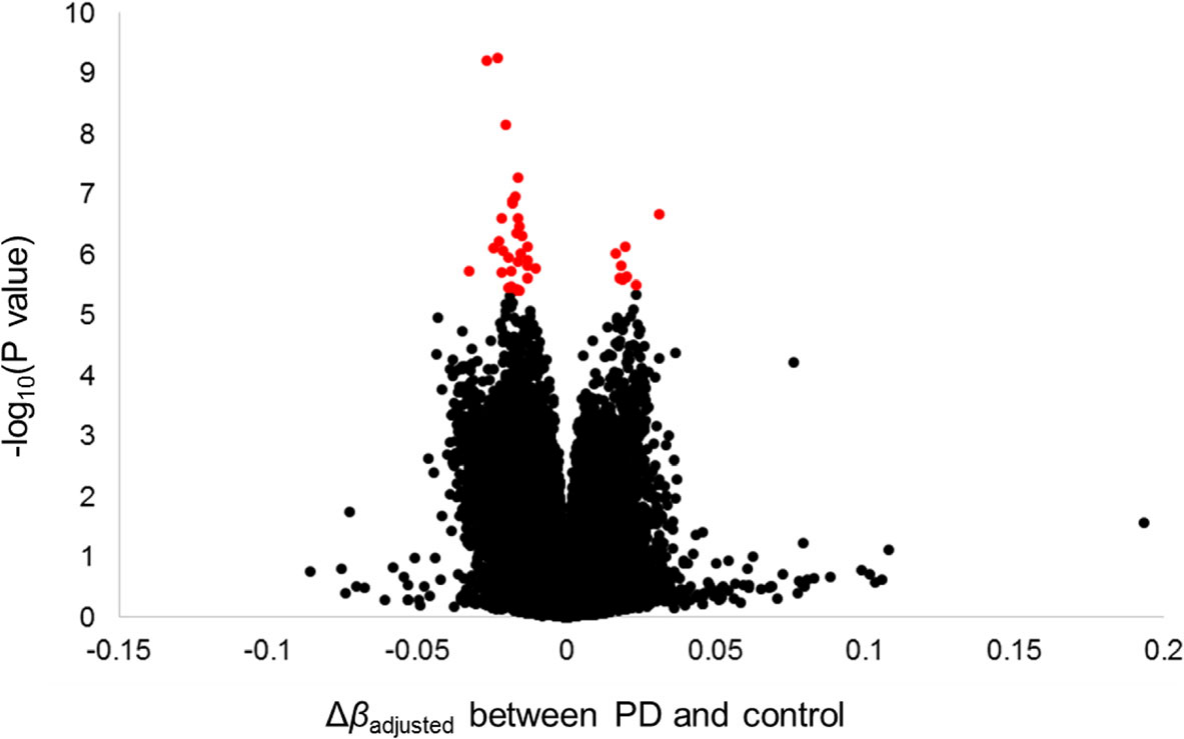
Results of the EWAS comparing between the PD and control subjects. Log-transformed *P* values of all the probes were plotted. The *horizontal axis* represents average adjusted *β* value differences (Δ*β*_adjusted_ = average *β*_adjusted (PD)_ − average *β*_adjusted (control)_) between PD and control subjects. Significant probes at 5% FDR correction are shown in *red dots*

With the DNA methylation array experiment, the methylation status of a total of 485,512 cytosine residues were examined. We filtered out the low quality probes and those on sex chromosomes, and finally, 376,602 probes remained (Additional file 1: Figure S5). The data were normalized via the pipeline, Lumi: QN + BMIQ + ComBat. We then performed an EWAS of PD. The Q-Q plot was showed in Additional file 1: Figure S6. After excluding three possible cross-reactive probes, 40 probes showed significant association with PD when the false discovery rate (FDR) was set to 5% (Table 1, Fig. 2). The most significant probe, cg25270498, was located upstream of the meteorin, glial cell differentiation regulator-like (*METRNL*) gene and was significantly hypomethylated in PD patients (FDR *q* value = 1.19 × 10^−4^), followed by cg05910615 (*HSPB6*; *C19orf55*, FDR *q* value = 1.19 × 10^−4^) and cg20340149 (*CLASP1*, FDR *q* value = 8.64 × 10^−4^). At many of the CpG sites with significantly different levels of DNA methylation, the cytosine residues were less methylated in the PD subjects than in the control subjects (Fig. 2). Only eight CpG sites were found to be significantly more methylated in the PD subjects than in the control subjects. Most of the significant CpG sites (37/40 CpG sites) were located in CpG island or CpG island shore and shelf that span up to 2 kb and 2–4 kb from CpG islands, respectively (Table 1). Many of them (27/40 CpG sites) were located upstream (within 1500 bp from the transcriptional start site, the 5′ untranslated region and the first exon) of genes, and these 27 CpG sites other than two were all hypomethylated in PD subjects (Table 1).

**Table 1:** Probes with significant differential DNA methylation status between PD and control

CHR	Position (hg19)	Target ID	Mean β value		Mean Adjusted M value				Genes	Location with respect to genes	Relation to CpG Islad^a^
			PD	Control	PD	Control	P value	q value			
17	81037414	cg25270498	0.257	0.283	-1.71	-1.53	5.67×10^-10^	1.19×10^-4^	*METRNL*	TSS200	Island
19	36248877	cg05910615	0.222	0.251	-2.05	-1.81	6.30×10^-10^	1.19×10^-4^	*HSPB6;C19orf55*	TSS1500;TSS200	N_Shore
2	122407145	cg20340149	0.152	0.175	-2.73	-2.49	6.88×10^-9^	8.64×10^-4^	*CLASP1*	TSS200	Island
13	80055594	cg14777817	0.134	0.153	-2.90	-2.69	5.17×10^-8^	4.87×10^-3^	*NDFIP2*	1stExon	Island
10	135088451	cg25526061	0.147	0.164	-2.79	-2.56	1.30×10^-7^	7.64×10^-3^	*ADAM8*	Body	N_Shore
17	27224823	cg04266864	0.133	0.151	-2.97	-2.73	1.42×10^-7^	7.64×10^-3^	*FLOT2;DHRS13*	TSS200;3'UTR	Island
16	12142335	cg10475689	0.606	0.576	0.82	0.63	2.09×10^-7^	9.38×10^-3^	*RUNDC2A*	Body	
16	23568708	cg05742564	0.192	0.215	-2.30	-2.08	2.48×10^-7^	9.38×10^-3^	*UBFD1;EARS2*	TSS200;TSS200	Island
1	228604037	cg02931001	0.263	0.280	-1.62	-1.49	2.49×10^-7^	9.38×10^-3^	*TRIM17*	5'UTR	Island
13	28024472	cg08209163	0.147	0.165	-2.73	-2.54	3.45×10^-7^	0.0118	*MTIF3*	TSS200;5'UTR	Island
12	50017361	cg10727759	0.147	0.165	-2.74	-2.54	4.51×10^-7^	0.0139	*PRPF40B*	TSS200	Island
22	24236284	cg12738349	0.290	0.304	-1.40	-1.30	4.80×10^-7^	0.0139	*MIF*	TSS1500	Island
3	197409980	cg08942682	0.830	0.848	2.06	2.28	6.05×10^-7^	0.0163	*KIAA0226*	Body	
6	170597377	cg05228964	0.602	0.580	0.71	0.59	7.23×10^-7^	0.0172	*DLL1*	Body	Island
12	4381997	cg08553284	0.316	0.330	-1.20	-1.11	7.57×10^-7^	0.0172	*CCND2*	TSS1500	Island
1	155164676	cg03425468	0.215	0.240	-2.10	-1.88	7.78×10^-7^	0.0172	*MIR92B*	TSS1500	Island
22	38202626	cg11029475	0.202	0.219	-2.23	-2.02	8.43×10^-7^	0.0176	*GCAT;H1F0;H1F0*	TSS1500;1stExon;3'UTR	N_Shore
16	2732724	cg02205746	0.295	0.311	-1.36	-1.26	9.40×10^-7^	0.0182	*KCTD5*	1stExon	Island
17	44270511	cg10256219	0.116	0.103	-2.71	-2.93	9.64×10^-7^	0.0182			Island
3	50375496	cg09386807	0.236	0.256	-1.86	-1.70	1.14×10^-6^	0.0204	*RASSF1*	TSS1500;Body;5'UTR	Island
8	28243934	cg13411962	0.168	0.181	-2.46	-2.31	1.25×10^-6^	0.0211	*ZNF395*	5'UTR;1stExon	Island
4	4861398	cg01959412	0.325	0.339	-1.17	-1.07	1.29×10^-6^	0.0211	*MSX1*	5'UTR;1stExon	Island
11	61197477	cg03342113	0.264	0.278	-1.58	-1.48	1.53×10^-6^	0.0230	*CPSF7;SDHAF2*	TSS200;TSS200	Island
17	42293627	cg24247482	0.556	0.557	0.50	0.39	1.53×10^-6^	0.0230	*UBTF*	Body	N_Shelf
17	80189962	cg17932802	0.358	0.366	-0.92	-0.85	1.65×10^-6^	0.0239	*SLC16A3*	TSS200;5'UTR	Island
1	204159498	cg13065121	0.233	0.250	-1.89	-1.73	1.84×10^-6^	0.0253	*KISS1*	3'UTR	N_Shore
6	32055370	cg26997880	0.225	0.247	-1.98	-1.79	1.95×10^-6^	0.0253	*TNXB*	Body	Island
12	121148158	cg19464320	0.123	0.107	-2.58	-2.83	2.26×10^-6^	0.0284	*UNC119B*	1stExon;5'UTR	N_Shore
9	87284706	cg13965062	0.230	0.243	-1.85	-1.75	2.39×10^-6^	0.0290	*NTRK2*	5'UTR;1stExon	Island
1	245316477	cg07124903	0.102	0.088	-2.88	-3.14	2.47×10^-6^	0.0290			N_Shore
19	11074303	cg08315613	0.574	0.570	0.59	0.48	2.54×10^-6^	0.0290	*SMARCA4*	5'UTR	S_Shore
2	242254519	cg13009927	0.196	0.214	-2.22	-2.05	3.28×10^-6^	0.0353	*SEPT2;HDLBP*	TSS1500;5'UTR	Island
2	217559020	cg03222971	0.233	0.251	-1.90	-1.73	3.42×10^-6^	0.0357	*IGFBP5*	Body	N_Shore
1	206223719	cg26795730	0.196	0.215	-2.23	-2.05	3.53×10^-6^	0.0357	*AVPR1B*	TSS1500	Island
2	73144353	cg15921587	0.302	0.318	-1.33	-1.21	3.69×10^-6^	0.0357	*EMX1*	TSS1500	Island
13	25621328	cg18098400	0.132	0.150	-2.96	-2.74	3.83×10^-6^	0.0357			Island
15	66993412	cg25048202	0.290	0.306	-1.41	-1.30	3.84×10^-6^	0.0357	*SMAD6*	TSS1500	N_Shore
2	27718181	cg04015759	0.250	0.267	-1.71	-1.58	3.89×10^-6^	0.0357	*FNDC4*	TSS200	Island
15	101690195	cg24378951	0.753	0.728	1.80	1.61	4.53×10^-6^	0.0406			
2	44059266	cg15889012	0.194	0.213	-2.25	-2.07	4.74×10^-6^	0.0415	*ABCG5*	Body	S_Shore

Abbreviation: CHR chromosome

TSS, transcription start site

UTR, untranslated region

^a^ Each category of “Relation to CpG island” column defines the following regions: Island, CpG island; N_Shore, 0-2 kb upstream of CpG island; S_Shore, 0-2 kb downstream of CpG island; N_Shelf, 2–4 kb upstream of CpG island

For confirmation, we examined the possibility that the significant associations were influenced by smoking status. We checked the distributions of adjusted *M* values of the significant CpG sites between smokers and non-smokers among the PD subjects and found that there was no effect of smoking for these sites in PD subjects (Additional file 1: Figure S6).

### Pathway analysis

To assess the overall influence of the significant differences in DNA methylation between the PD and control subjects, a pathway analysis was performed. Annotation information on the 40 significantly associated CpG sites (Table 1) was used for the analysis; in total, 42 genes were annotated to the CpG sites. Three gene sets showed significant associations at a FDR of 5% (Table 2) after we excluded pathways that showed no association in the GOseq pathway analysis in which gene length was taken into consideration. The identified pathways included the “positive regulation of lymphocyte activation” gene set.

**Table 2:** Result of the pathway analysis

Gene sets	Number of genes in pathways	*P* value	FDR	Number of genes in the data	Associated genes in the data
Epidermis development	426	1.5 × 10^−4^	0.017	5	*DLL1*, *FLOT2*, *IGFBP5*, *MIF*, *SMARCA4*
Positive regulation of cell cycle	493	3.3 × 10^−4^	0.019	5	*CCND2*, *AVPR1B*, *MIF*, *RASSF1*, *MSX1*
Positive regulation of lymphocyte activation	469	1.9 × 10^−3^	0.037	5	*MIF*, *ADAM8*, *IGFBP5*, *AVPR1B*, *FLOT2*

*FDR* false discovery rate

## Discussion

In this study, we performed an EWAS of PD using a DNA methylation array and examined the genome-wide DNA methylation profiles of PD for the first time, as far as we know, although replication is necessary in future studies. This array technology can target 99% of genes and 95% of CpG island regions [31] and enables us the analysis of DNA methylation status in a genome-wide manner [32]. Recently, a number of studies have employed this platform to identify differentially DNA methylation sites according to phenotypes. In particular, in the cancer field, this platform has been used to identify a number of DMPs accompanying large *β* value differences in cancer cells (≥0.2) [28]. DNA methylation is considered to change according to environmental factors [33, 34]; as such, in psychiatric disorders, it was predicted that the DNA methylation levels at specific sites would differ between patients and healthy subjects [12]. However, in the present study, no DMP showed a large *β* value difference (≥0.2) between the PD and control subjects.

In the psychiatric field, epigenetics has been considered to play a role in disease pathogenesis and several recent studies have examined the relationships between DNA methylation and psychiatric disorders in a genome-wide manner. For example, an EWAS of major depressive disorder identified more than 350 CpG sites that were associated with the disease and all of these CpG sites were hypomethylated in the major depressive disorder patients8 [35] Other EWASs of suicidal behavior or early life stress-associated depression found that the DNA methylation status differed globally between the patients and control subjects [36–38]﻿. In addition, EWASs of schizophrenia identified numerous DMPs associated with the disease; a part of these DMPs were annotated in gene regions previously reported as candidate genes of schizophrenia or psychiatric diseases [39–42]. The results of these previous studies suggest the possibility that in psychiatric disorders, multiple DMPs with small effects (*β* value difference ≤ 0.1) might be associated with diseases together. This would be one explanation for an inflation of *P* values of the regression analysis observed in this study (Additional file 1: Figure S6), although it cannot be denied that several factors other than age, sex ,and proportions of leukocyte subsets potentially confounded the result.

In this study, 40 CpG sites were found to be significantly associated with PD. Among these, 27 CpG sites were located upstream of genes and with the exception of two CpG sites, they were all hypomethylated in PD subjects when compared with control subjects. According to previous studies, DNA hypomethylation of upstream gene regions is often associated with a higher level of gene expression [11, 43, 44]. Therefore, such DNA methylation differences upstream of genes may be related to a higher expression level of the annotated genes. We further performed pathway analyses to evaluate the overall influence of the DNA methylation differences. Among the detected gene sets, “positive regulation of lymphocyte activation,” which reflected the significant probes annotated to *MIF* or *ADAM8*, *IGFBP5*, *AVPR1B*, and *FLOT2*, was particularly intriguing. We previously reported the associations of the immune pathways and the specific *HLA* allele, HLA-DRB1*13:02, with PD [45]. Furthermore, the most significant probe, cg25270498, located upstream of *METRNL*, which was reported to possibly act as a cytokine and to exert effects on immune process [46].

In the current study, we also examined the DNA methylation around candidate genes of which DNA methylation statuses were previously reported to be associated with anxiety disorders. The gene regions of *OXTR*, *GAD1*, *SLC6A4*, *SLC6A2*, and *MAOA* were individually examined. As a result, with our sample set, no significant differences between PD and healthy subjects were observed in these candidate gene regions (Additional file 2: Tables S1–S5). None of the genes annotated to the significant CpG sites in this study have been identified in previous studies of PD, including GWASs [8, 47]. This might have been partly due to the relatively small sizes of the samples analyzed, or because the CpGs examined using the array were not sufficient in density. Therefore, future detailed studies with larger samples are necessary to further investigate the relationship between the PD candidate genes and DNA methylation.

The proportions of leukocyte subset estimated with the DNA methylation array data were compared between the PD and healthy subjects in this study. As a result, a higher proportion of CD4^+^ T cells (*P* = 0.0034) was found in the PD subjects. A higher tendency of the abundance measure of naive CD4^+^ T cell was also observed in PD subjects. As major histocompatibility complex class II molecules, including HLA-DR, interact mainly with CD4^+^ T cells, an increase in CD4^+^ T cells might be a part of an immune abnormality in PD patients. However, results of previous studies on surface immune phenotypes of lymphocytes did not report consistent results on the up- and down-regulation of leukocyte subsets [48–51]. Additionally, lymphocytes can be affected by environmental factors and/or infection status. Further, the comparisons for leukocyte subsets in this study were not based on cell count or abundance and they were not independent; therefore, further analysis of surface markers by flow cytometry with larger samples is needed to validate the association of CD4^+^ T cells with PD.

There are several limitations to this study: lack of validation and replication studies, use of blood, potential confounding of other factors, and small sample size. First, the current study lacks replication analysis using other DNA methylation measurement such as pyrosequencing. Although replication with pyrosequencing makes the results more reliable, almost all the significant CpG sites in this study are located in or around CpG islands. Consequently, it is difficult to design the primers for pyrosequencing. Second, we used DNA extracted from peripheral blood rather than brain samples. Several studies have reported that disease-associated DNA methylation abnormalities can be detected across tissues [39, 42, 52, 53], but there are clear tissue-specific differences in DNA methylation profiles [54, 55]. We checked the blood-brain correlations of the significant CpG sites of this study, using Blood Brain DNA Methylation Comparison Tool [55]. We found that nine out of 40 significant CpG sites showed the blood-brain correlation >0.3, in at least one region of the four examined brain regions (Additional file 3: Table S6). However, we consider that there is a possibility that biological processes in blood such as immune abnormalities are associated with PD [45]. A previous study of multiple sclerosis, an autoimmune disease of the central nervous system, has reported an association of DNA methylation status of a CpG site in blood with the disease [56], supporting our hypothesis. Nevertheless, detailed studies using brain samples are needed to find additional and/or tissue-specific DNA methylation differences associated with PD. Moreover, DNA methylation was found to be influenced by SNP genotypes [55, 56]. The DNA methylation status of a CpG site in multiple sclerosis, which we have mentioned above, is also affected by SNP genotypes [56]. As for the 40 significant probes, two probes, cg07124903 and cg20340149, have previously been reported to be methylation QTLs (meQTLs) in developing brain and are influenced by genetic variations (Additional file 3: Table S7) [57]. We also checked meQTLs identified with blood samples and found that only one probe cg07124903 was reported to be meQTLs (Additional file 3: Table S7) [58]. Additional studies combining the GWAS SNP data and EWAS data might provide new knowledge on the relationships among DNA methylation, SNPs, and PD. Finally, we were unable to take into account the effects of medication for PD and smoking. A previous study reported that long-term medication for schizophrenia decreased DNA methylation of the *GAD1* promoter, which was hypermethylated in a mouse model of schizophrenia [59]. Most of the PD patients in the present study were prescribed psychotropic medications. As such there is a possibility that the drugs affected the DNA methylation differences between the PD and healthy subjects. In addition, some DNA methylation sites have been reported to be influenced by smoking [60]. In this study, we could not adjust for such smoking effects as we did not have data on the smoking status of the healthy control subjects. However, when we examined the distributions of *M* values of the significant CpG sites between smokers and non-smokers among the PD subjects, we found that there was no effect of smoking for these sites (Additional file 1: Figure S8).

In conclusion, there might not be any CpG sites with DNA methylation differences that have a large effect on PD. However, we obtained some intriguing results: the hypomethylated CpG sites annotated to genes associated with the leukocyte activation pathway and the higher proportion of CD4^+^ T cells in PD. There is a possibility that several CpG sites with small effects, especially those that are related to immunity, are associated, together as a group, with PD. Further replication studies with larger number of samples are necessary to confirm the findings of this study.

## Conclusions

We performed the EWAS of PD and identified 40 CpG sites of which the levels of DNA methylation were significantly different between PD and healthy control subjects. Some of these CpG sites have the possibility to be related the “positive regulation of lymphocyte activation” pathway. Such CpG sites with small effects might be associated, together as a group, with PD.

## Methods

### Subjects

DNA samples for the EWAS were obtained from our PD and healthy control sample set: patients with PD (*N* = 48) and age- and sex-matched healthy control subjects (*N* = 48) were recruited from among Japanese individuals living in Tokyo and Nagoya, located in the center of mainland Japan (Additional file 4: Table S8. These samples other than one discordant monozygotic twins were from unrelated PD patients and healthy control subjects. Each PD patient was diagnosed according to the Diagnostic and Statistical Manual of Mental Disorders, 4th Edition (DSM-IV) criteria [61] based on responses to the Mini International Neuropsychiatric Interview (MINI) [62] and clinical records. Healthy control subjects were interviewed by psychiatrists and were asked to fill out a questionnaire, MINI, in order to exclude those with a history of a major psychiatric illness, including PD.

### Epigenome-wide DNA methylation analysis

Genomic DNA (PD, *N* = 48; control, *N* = 48) was extracted from leukocytes in whole blood by the standard phenol chloroform method (Wizard genomic DNA purification kit, Promega Corporation, WI, USA). DNA samples were first bisulfite-converted using a kit for the bisulfite conversion of DNA (EZ DNA Methylation™ Kit, Zymo Research, Irvine, CA, USA). For all samples, the DNA methylation levels of cytosine residues across the genome were examined with a DNA methylation array (Infinium® Human Methylation 450K BeadChip, Illumina Inc.) according to the manufacturer’s protocol. Briefly, the bisulfite-converted DNA samples underwent whole-genome amplification and were fragmented and hybridized on BeadChip. After hybridization of the fragmented DNA with their complementary probe sequences, the DNA methylation status was determined through a single-base extension step. The arrays were imaged with a high-precision scanner (iScan system, Illumina Inc.), and the signal intensities were extracted using a software package (GenomeStudio Software, Illumina Inc.). The DNA methylation status of each cytosine residue was evaluated with the *β* value, which is the ratio of the signal from the methylated probe divided by the total signal intensity. The *β* value ranges from 0 (unmethylated) to 1 (completely methylated).

### Data filtering and normalization

Data filtering and processing were performed for quality control of the calculated *β* values. *β* values with a detection *P* value <0.01 were treated as missing values. We then calculated the ratio of the detected *β* values to all of the examined *β* values (*N* = 96) for each probe; this was defined as the probe call rate. Probes that met the following conditions were used in the subsequent analyses: (1) probe call rate >95%; (2) probe not on a sex chromosome; (3) probe not including a single-nucleotide polymorphism (SNP) with a minor allele frequency ≥0.05; and (4) probe not reported to be cross-reactive [63] (Additional file 1: Figure S5). As mentioned in the last criterion, we excluded cross-reactive probes that were reported to co-hybridize to alternate sequences that are highly homologous (<4 base mismatches among 50 bases) to the intended targets [63]. Furthermore, we created a list of possible cross-reactive probes that have unintended target sequences identical to the 20-base sequence from the 5′ end of each intended target (Additional file 5: Table S9). The 20 bases from the 5′ end of each target were mapped against the reference sequence (Genome Reference Consortium Human Reference 37 (GCA_000001405.1)) using BLAST (https://blast.ncbi.nlm.nih.gov/Blast.cgi). In examining significant probes, we excluded the probes that were on the list.

After the filtering, data normalization was performed, with the following pipeline, Lumi: quantile normalization (QN; correction for the distributions of the pooled probes) + beta-mixture quantile (BMIQ) normalization (correction for probe design bias) + correction for the batch effect (ComBat). First, the distributions of the pooled methylated and unmethylated probes were quantile normalized using the Lumi package under the assumption that they were similar between different samples [64]. A beta-mixture QN method was used to correct probe design bias with BMIQ normalization [65]. Finally, an empirical Bayes batch-correction method, ComBat [66], was employed to control for batch effects among arrays. In order to detect PD-associated DMPs, the original *β* values were converted to *M* values via the logit transformation [67] and used for performing the case control analysis. As for probes on X chromosome, the data filtering and normalization were performed in the same way separately only with the female samples (PD, *N* = 31, control, *N* = 31) (Additional file 1: Figure S8).

### Prediction of the DNA methylation age and the distributions of leukocyte subsets

DNA methylation age (DNAm age) was defined as the age estimated from the DNA methylation status data of several CpG sites. We predicted the DNAm age to evaluate the reliability of the assay in both PD and healthy control subjects. DNAm age was estimated using the EWAS data following the algorithm reported in a previous study [24]. As for the analysis of the estimated DNAm age, the data were normalized according to the previous report [24], because the probes used in this analysis were a portion of the total probes and they did not include any type II probe. The estimated DNAm age was compared with chronological age by calculating Pearson’s correlation coefficients.

Furthermore, DNA methylation data were used to predict the cell mixture distributions of leukocyte subsets [30] to examine the possibility that cell mixture distributions differ between PD and healthy subjects. The proportions of leukocyte subsets (natural killer cells, B cells, CD4^+^ T cells, CD8^+^ T cells, monocytes, and granulocytes) were estimated using a published algorithm [30] with an R package, Minfi. Briefly, the *β* values of CpG sites, which correspond to putative differentially methylated sites among leukocyte subsets and that enable them to be distinguished, were selected. The selected *β* values were applied to the analysis, which resembled a regression calibration, as it can be considered a surrogate measure of the distribution of leukocyte cell mixtures [30]. The estimated proportions of leukocyte subsets were compared between the PD and control subjects. Additionally, we estimated abundance measures of plasmablasts, CD8^+^CD28^−^CD45RA^−^ T cells, naive CD8^+^ T cells, and naive CD4^+^ T cells using the epigenetic clock software [24].

### Pathway analysis

A pathway analysis was performed using the MetaCore™ platform (version 6.24 build 67895, Thomson Reuters, New York, NY, USA). Genes annotated to significant CpG sites were examined to determine whether they had any enrichment of gene sets for biological processes and molecular functions in the GO database (http://geneontology.org/) [68]. To be more precise, if the significant CpG sites were located in regions within 1500 bp from a transcription start site, 5′ UTR, body, and 3′ UTR of genes, the genes were annotated to the CpG sites and included in the pathway analysis. Since gene sets with large numbers of genes have a tendency to represent broader categories and have no useful biological meaning, gene sets with more than 500 genes were disregarded [69]. Gene sets with less than five registered genes or five consequent genes annotated from a list of examined genes were also disregarded because such gene sets are worthy of little attention in a pathway-based approach. Furthermore, we also used another method of GO-based pathway analysis, GOseq [70], in which bias caused by the different numbers of probes associated with each gene can be corrected [70, 71]. Pathways identified using the MetaCore™ platform, but not replicated with GOseq analysis, were excluded from the list of significant pathways.

### Statistical analysis

The Wilcoxon rank sum test was employed to compare the proportions of the leukocyte subsets between the PD and control subjects. The Bonferroni correction was applied to adjust for multiple comparisons.

For the EWAS, significant associations were assessed by linear regression analysis with adjustments for the effects of the predicted proportions of leukocyte subsets using *M* values at a false discovery rate (FDR) of 5%. To check the effect of smoking on the significant sites, adjusted *M* values were compared between smokers and non-smokers in PD group using *t* test.

All analyses were performed using R software.

## Additional files

Additional file 1: Figures S1–S8. Figures which were used for the quality check of the DNA methylation array data, filtering procedure, and additional leukocyte subset analysis and check for smoking effect are indicated. (PDF 727kb)
Additional file 2: Tables S1–S5. Results of probes in the gene regions of which DNA methylation were previously reported to be associated with anxiety disorders. (PDF 146 kb)
Additional file 3: Table S6 and S7. Blood-brain correlations of the significant CpG sites and meQTL sites found in the significant CpG sites. (PDF 40 kb)
Additional file 4: Table S8. Demographic characteristics of samples. (PDF 34 kb)
Additional file 5: Table S9. Probe IDs of possible cross-reactive probes. (XLSX 446 kb)
