# Supplementary material for: Epigenome-wide association study of DNA methylation in panic disorder
Source: Clin Epigenetics. 2017 Jan 21;9:6. doi: 10.1186/s13148-016-0307-1 (PMC5270210; doi:10.1186/s13148-016-0307-1)
Supplement: Additional file 1: Figures S1–S8. — Figures which were used for the quality check of the DNA methylation array data, filtering procedure, and additional leukocyte subset analysis and check for smoking effect are indicated. (PDF 727kb) [file 13148_2016_307_MOESM1_ESM.pdf]

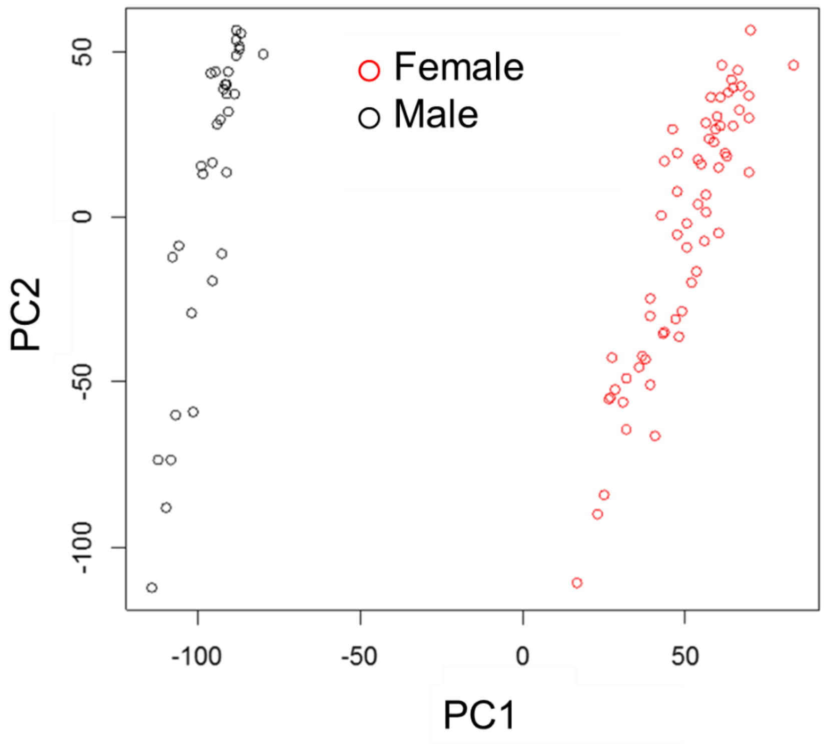

Figure S1. Result of the PCA checking gender distribution

The PCA using probes on the X chromosome was performed to examine whether the samples were correctly labeled with the presumed gender. Red dots indicate female samples and black dots indicate male samples. All samples were classified into the presupposed gender groups.

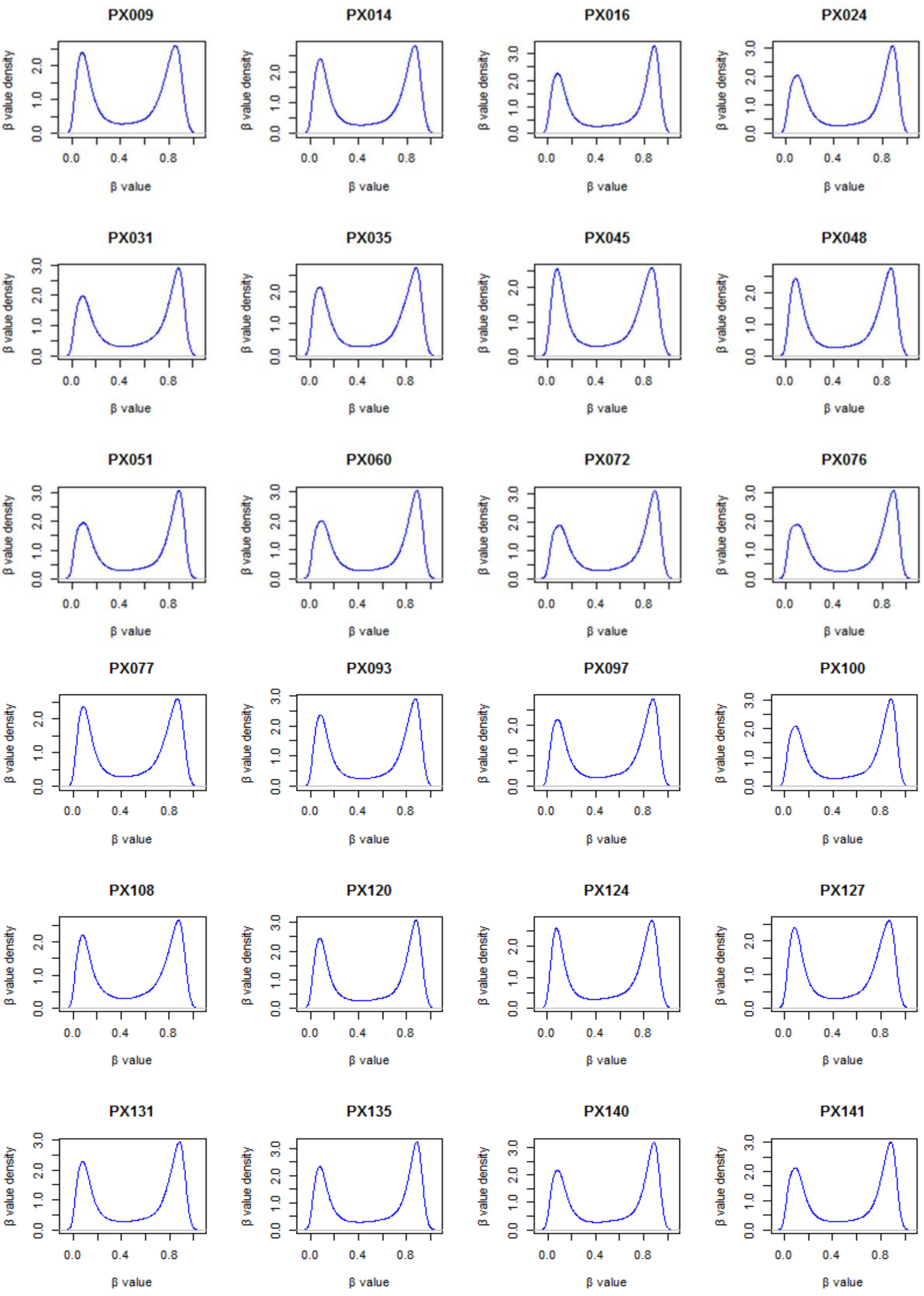

Figure S2. The density plot of each sample's β values (1/4)

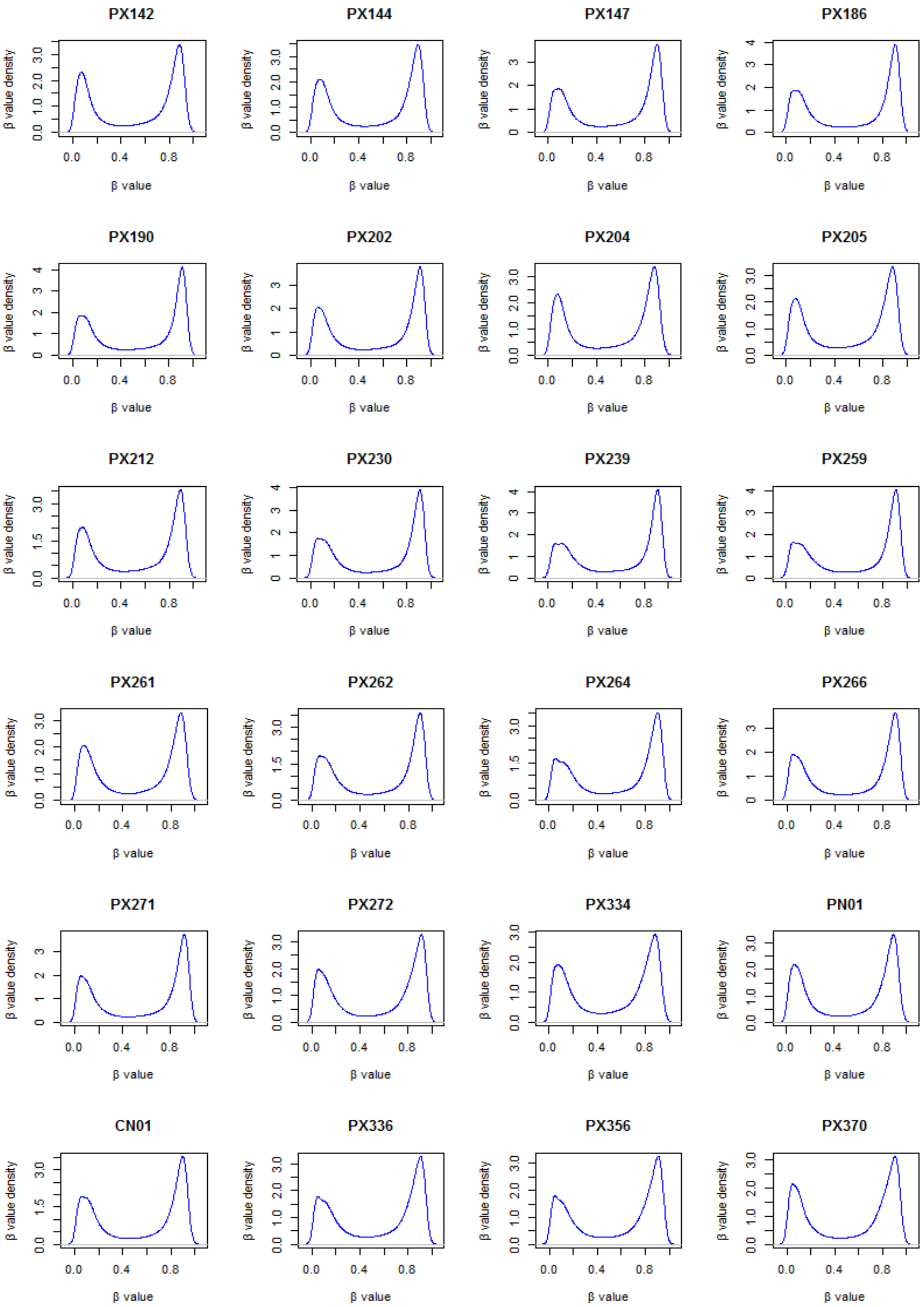

Figure S2. The density plot of each sample's β values (2/4)

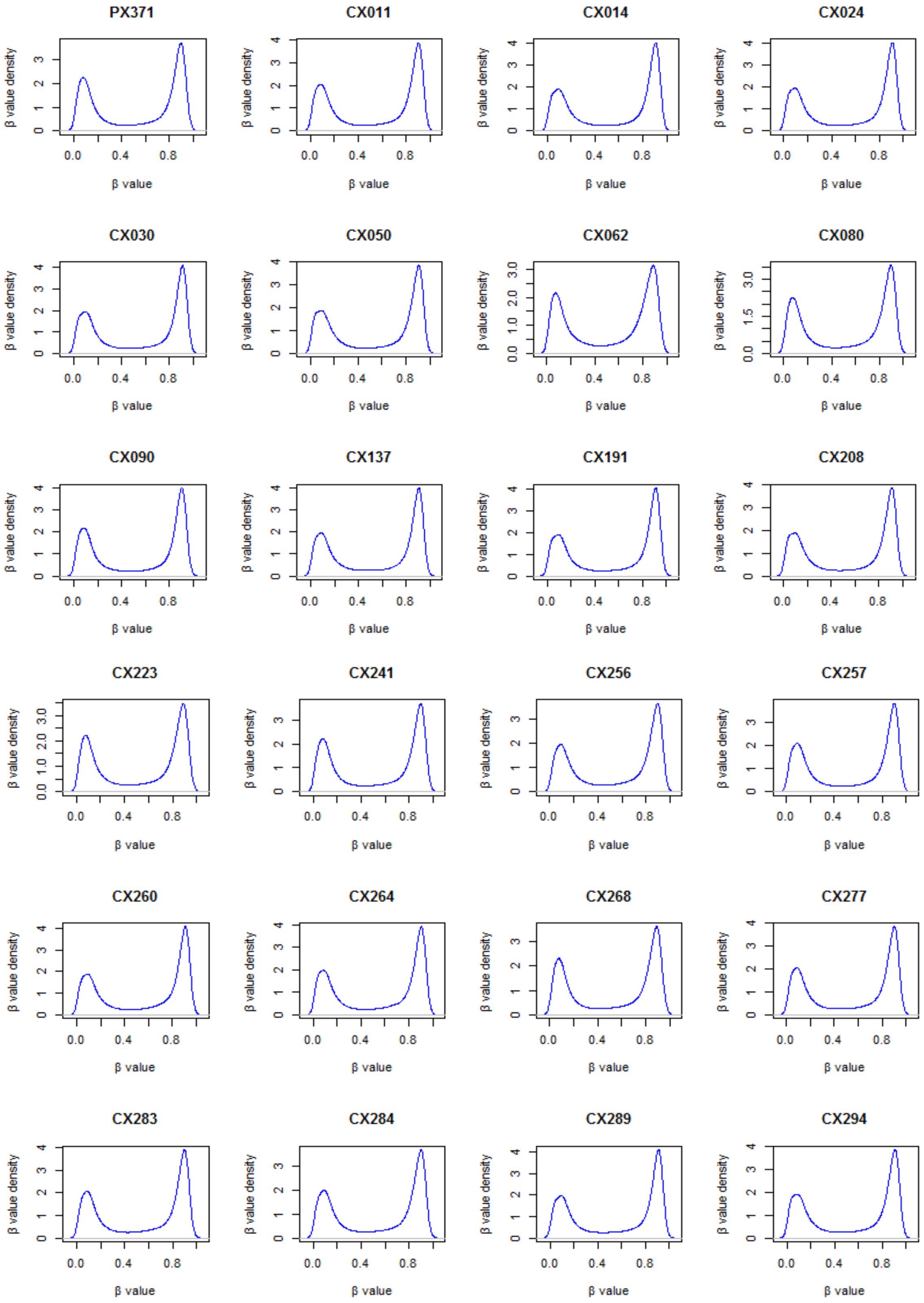

Figure S2. The density plot of each sample's β values (3/4)

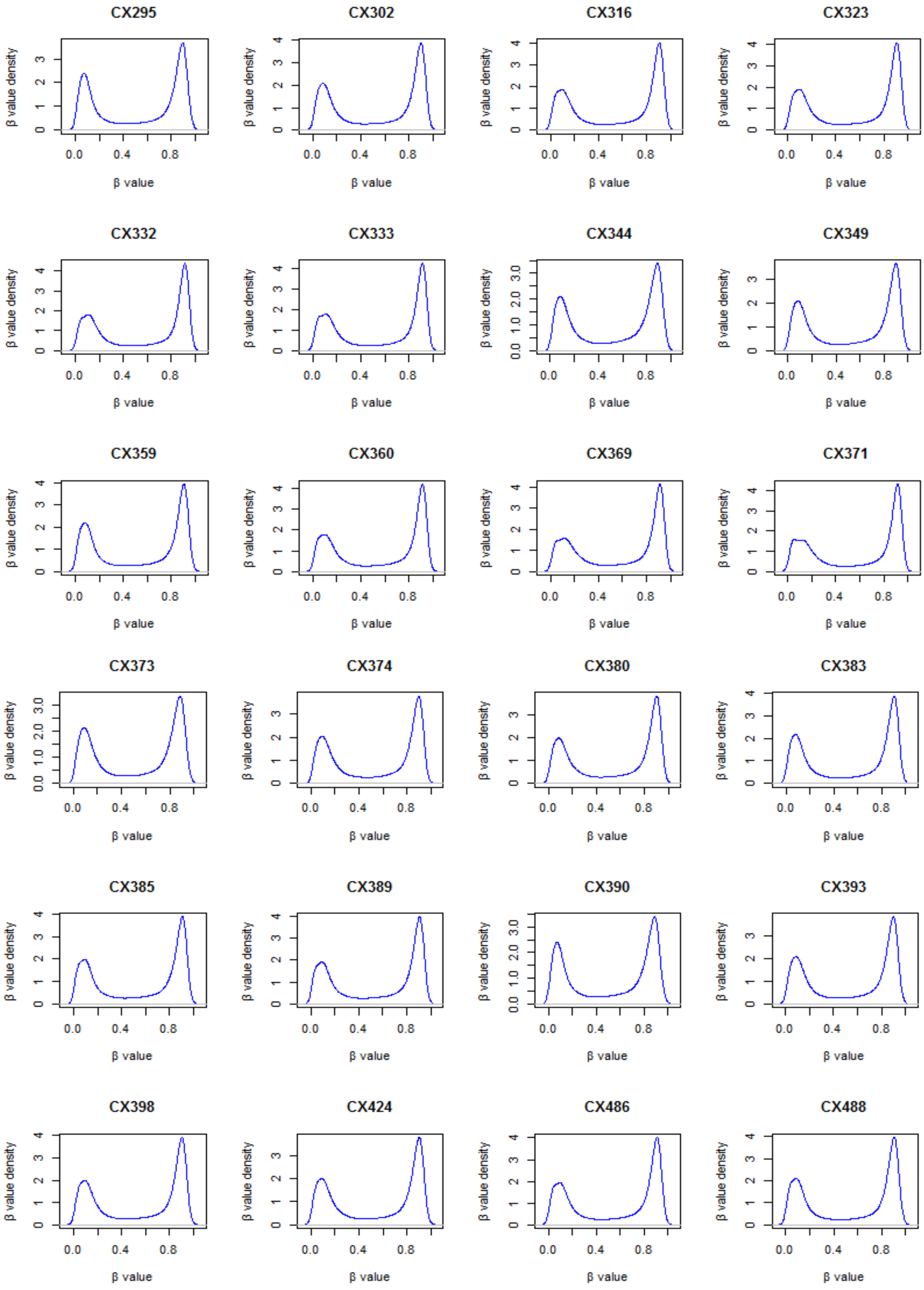

Figure S2. The density plot of each sample's β values (4/4)

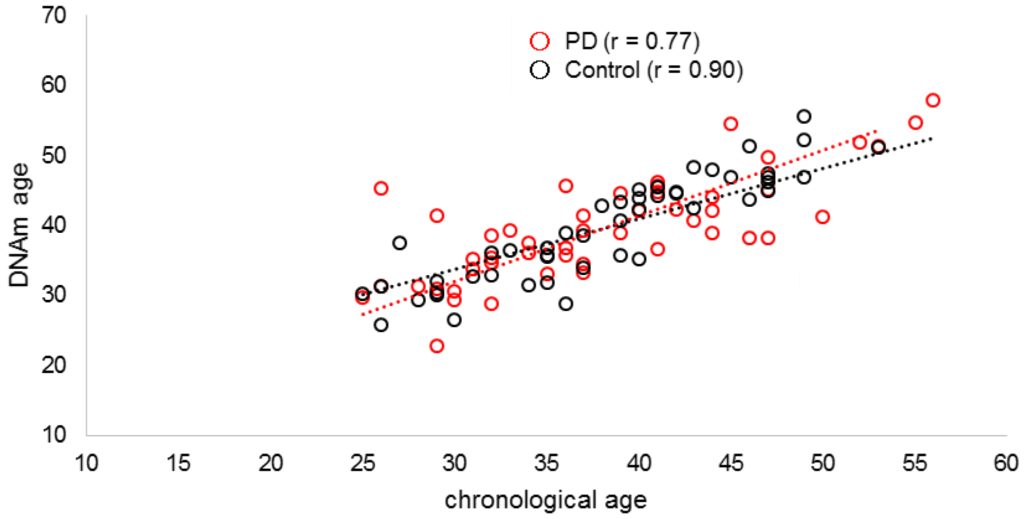

Figure S3. Correlation between DNAm age and chronological age

DNAm age was predicted using the results of the methylation array. Red and black dots indicate each PD and healthy control subjects respectively. Pearson's correlation coefficients between DNAm age and chronological age were calculated as 0.77 and 0.90 in each group.

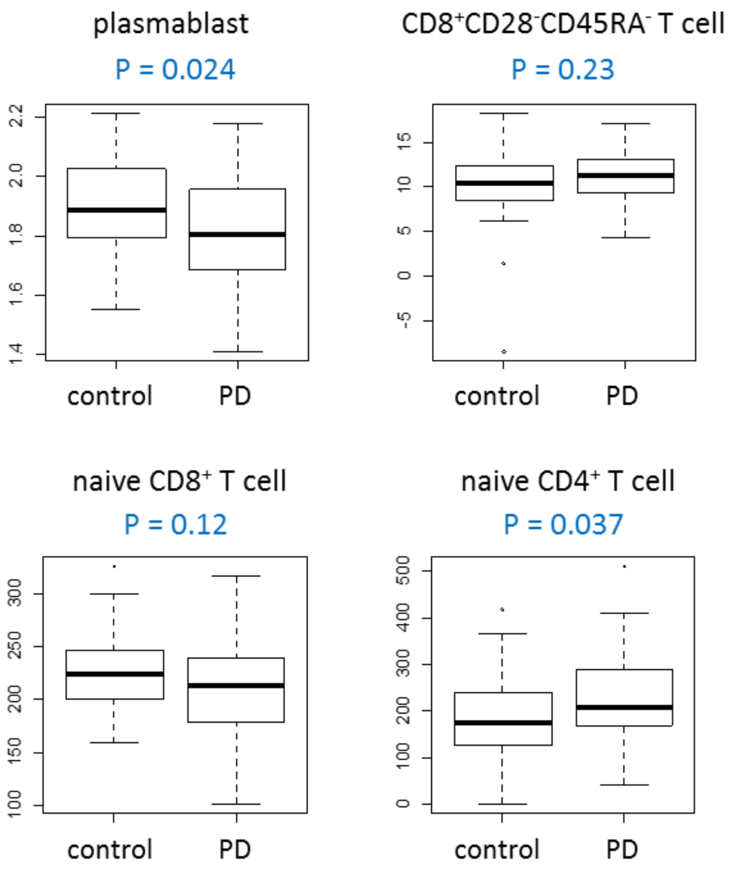

Figure S4. The estimated abundance measures of plasmablasts, $CD8^+CD28^-CD45RA^-$ T cells, naive $CD8^+$ T cells, and naive $CD4^+$ T cells

The abundance measures were estimated using the results of the DNA methylation array. Wilcoxon rank-sum tests using the estimated abundance measures were performed between the PD and control subjects. P values are indicated in blue characters. Significance level after the Bonferroni correction was set as $\alpha = 0.005$.

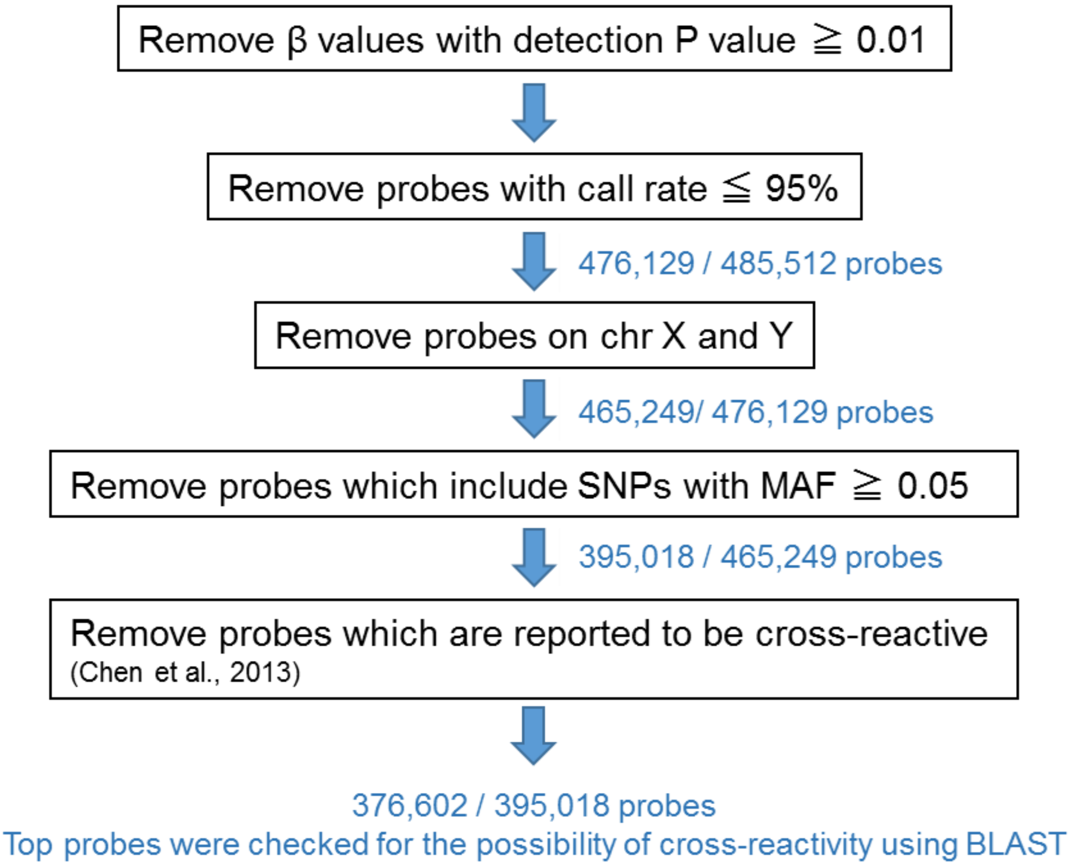

Figure S5. Filtering procedure of the methylation array data

β values with detection P value < 0.01 were treated as missing values and then probe call rates were determined. Low quality probes were filtered out applying the following criteria; (1) probe call rate > 95%, (2) probes not on chromosome (Chr.) X and Y, (3) probes not including SNPs with MAF ≧ 0.05, (4) probes not reported as cross-reactive. Finally 376,602 probes remained and were examined in the following analyses.

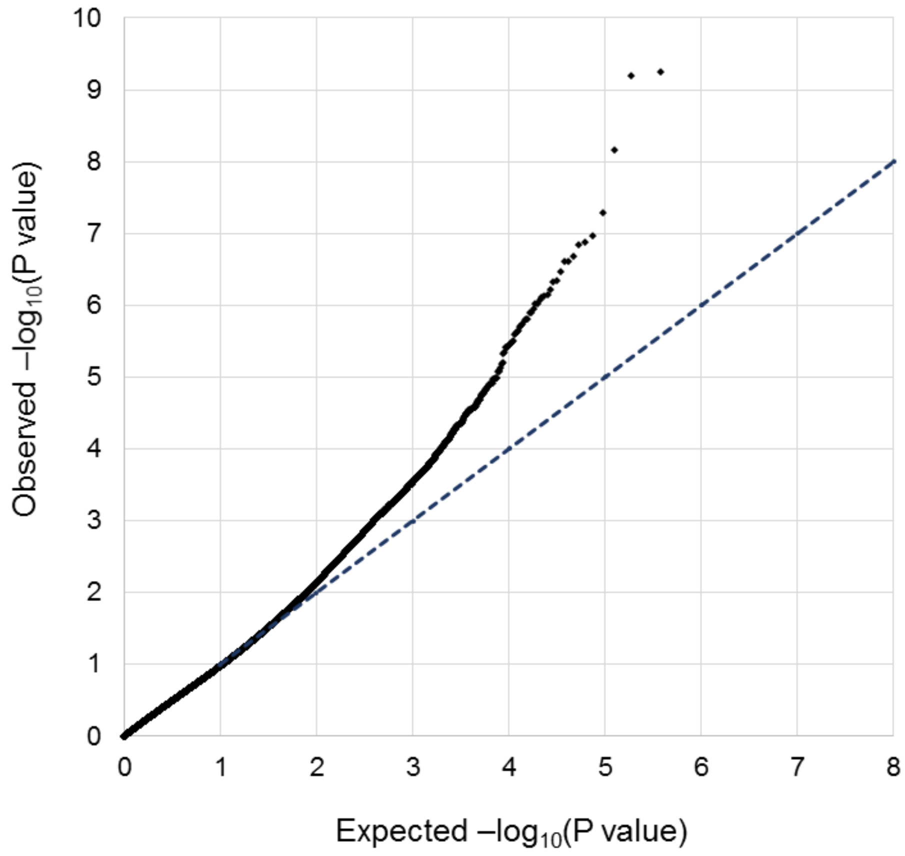

Figure S6. Q-Q plot of regression analysis
The observed P values are those of regression analysis adjusting the effects of the predicted proportions of leukocyte subsets.

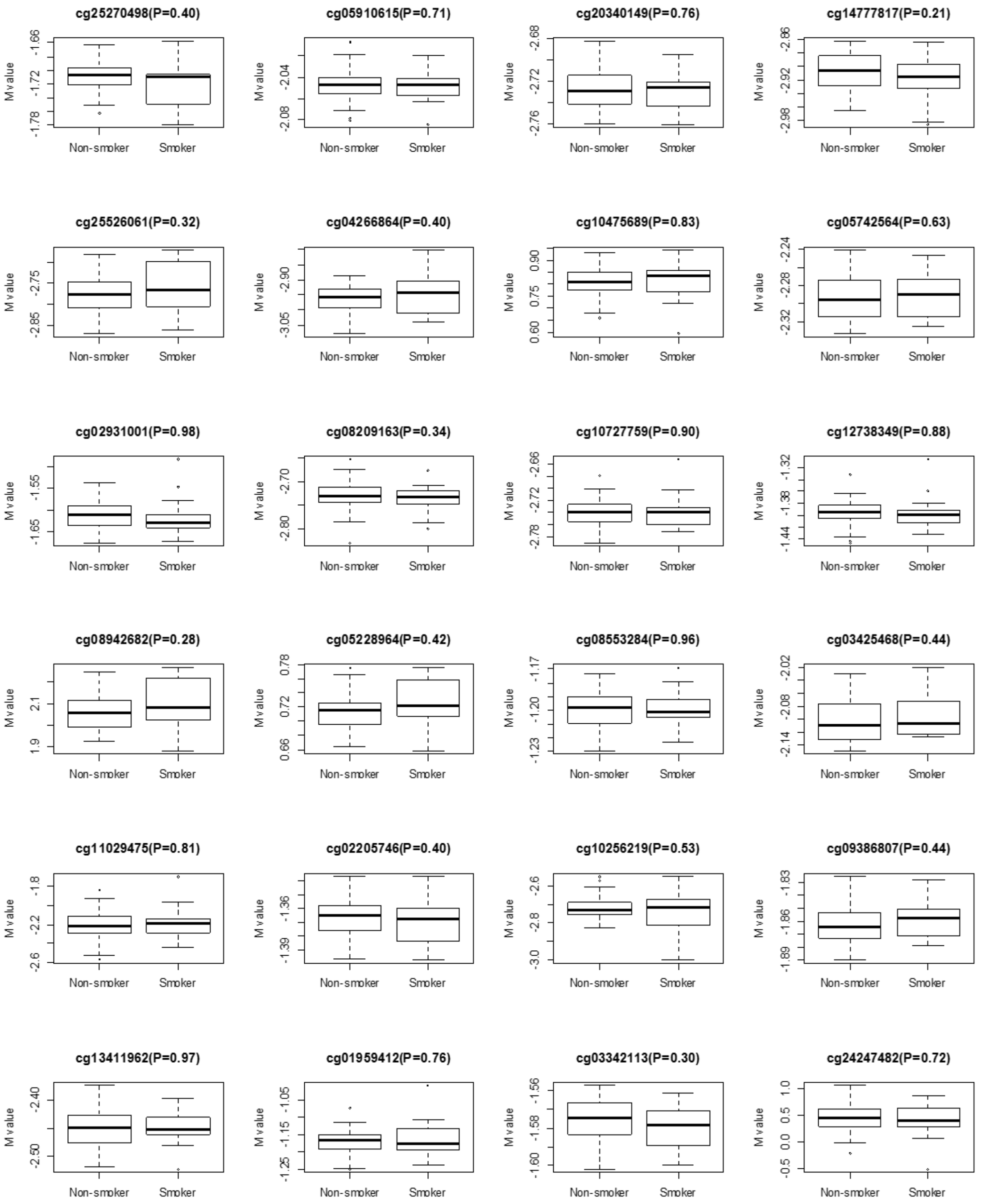

Figure S7. The M value distributions of the significant CpG sites between smokers and non-smokers in PD subjects (1/2)

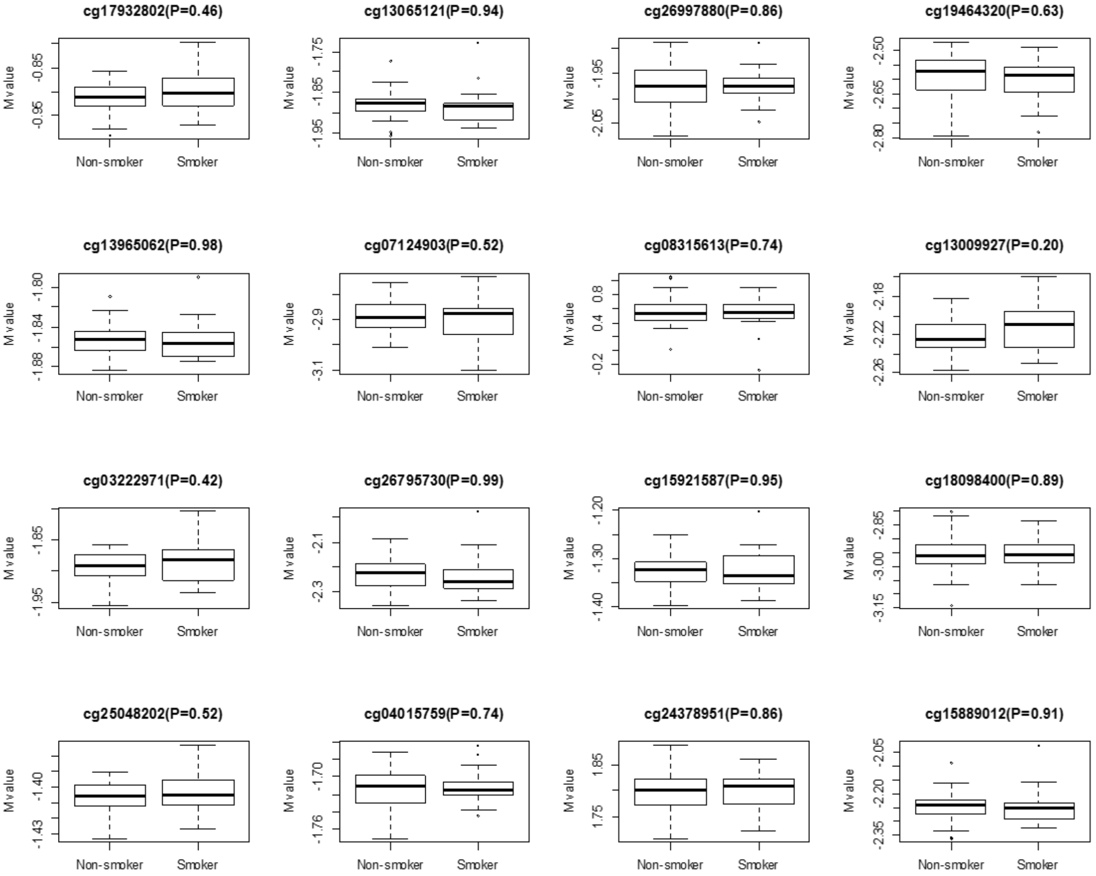

Figure S7. The M value distributions of the significant CpG sites between smokers and non-smokers in PD subjects (2/2)

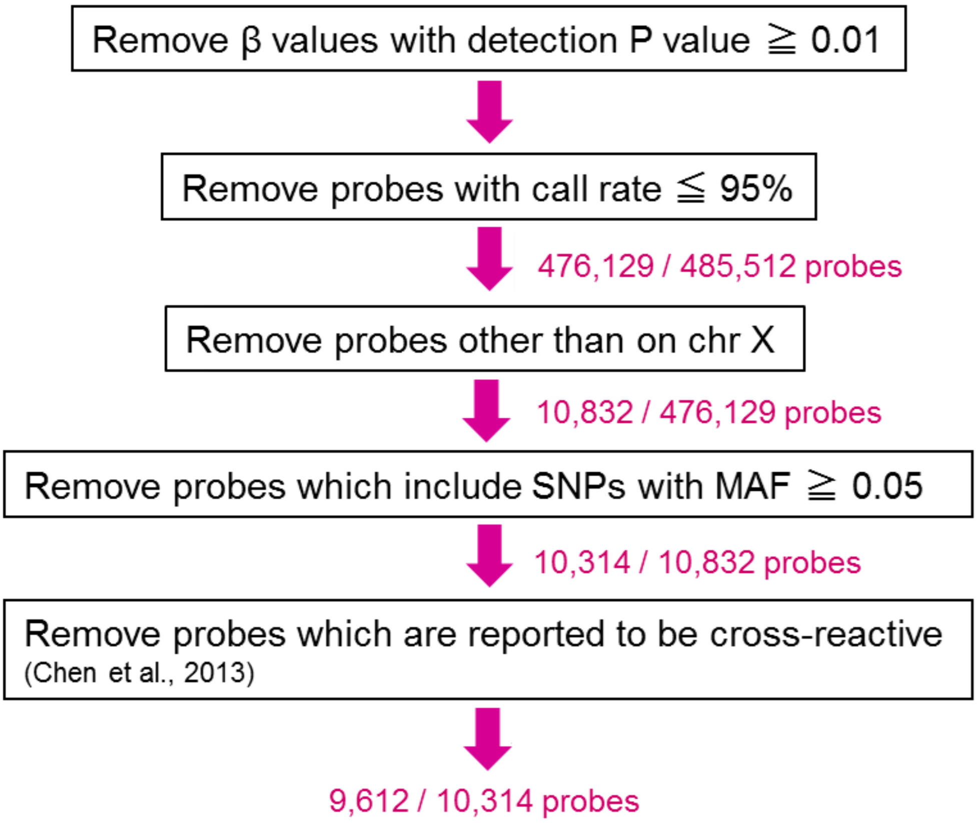

Figure S8. Filtering procedure of probes on X chromosome

β values with detection P value < 0.01 were treated as missing values and then probe call rates were determined. Low quality probes were filtered out applying the following criteria; (1) probe call rate > 95%, (2) probes on chromosome (Chr.) X, (3) probes not including SNPs with MAF ≧ 0.05, (4) probes not reported as cross-reactive. Finally 9,612 probes remained and were examined in the following analyses.
