# Supplementary material for: Epigenome-wide association study of DNA methylation in panic disorder
Source: Clin Epigenetics. 2017 Jan 21;9:6. doi: 10.1186/s13148-016-0307-1 (PMC5270210; doi:10.1186/s13148-016-0307-1)
Supplement: Additional file 2: Tables S1–S5. — Results of probes in the gene regions of which DNA methylation were previously reported to be associated with anxiety disorders. (PDF 146 kb) [file 13148_2016_307_MOESM2_ESM.pdf]

Table S1. Results of probes in the *OXTR* region on chromosome 3

Target ID	Position (hg19)	Mean β value		Mean Adjusted M value		P value	Location with respect to *OXTR*	Relation to CpG Islad[a]
		PD	Control	PD	Control			
cg15317815	8809306	0.201	0.204	-2.11	-2.07	0.763	Body	Island
cg04523291	8809501	0.176	0.171	-2.30	-2.33	0.816	Body	Island
cg02192228	8809536	0.165	0.164	-2.38	-2.40	0.873	Body	Island
cg27501759	8809715	0.095	0.092	-3.40	-3.45	0.768	Body	Island
cg12695586	8810077	0.047	0.048	-4.36	-4.37	0.907	5'UTR	Island
cg19619174	8810139	0.049	0.048	-4.25	-4.31	0.438	5'UTR	Island
cg03987506	8810549	0.068	0.068	-3.85	-3.84	0.875	5'UTR	Island
cg08535600	8810980	0.093	0.097	-3.38	-3.31	0.242	1stExon;5'UTR	Island
cg17285225	8811004	0.083	0.084	-3.54	-3.50	0.465	1stExon;5'UTR	Island
cg09353063	8811092	0.115	0.121	-2.95	-2.97	0.865	1stExon;5'UTR	Island
cg23391006	8811279	0.035	0.035	-4.88	-4.87	0.914	1stExon;5'UTR	Island
cg00247334	8811543	0.825	0.832	2.27	2.30	0.699	TSS1500	S_Shore
cg17036624	8811601	0.487	0.506	-0.21	-0.08	0.082	TSS1500	S_Shore
cg14483142	8811758	0.776	0.780	1.85	1.84	0.960	TSS1500	S_Shore

Abbreviation: TSS, transcription start site
UTR, untranslated region

[a] Each category of "relation to CpG island" column defines the following regions: Island, CpG island; S_Shore, 0-2kb downstream of CpG island.

Table S2. Results of probes in the *GAD1* region on chromosome 2

Target ID	Position (hg19)	Mean β value		Mean Adjusted M value		P value	Location with respect to *GAD1*	Relation to CpG Islad[a]
		PD	Control	PD	Control			
cg03443455	171671795	0.262	0.265	-1.54	-1.51	0.507	TSS1500	Island
cg03448612	171672694	0.032	0.031	-4.94	-4.97	0.699	TSS1500	Island
cg09742688	171672899	0.025	0.024	-5.28	-5.33	0.369	TSS1500	Island
cg23221504	171673110	0.103	0.102	-3.13	-3.15	0.773	TSS200	Island
cg00915206	171673199	0.019	0.018	-5.62	-5.72	0.236	5'UTR;1stExon	Island
cg11582100	171673207	0.031	0.028	-4.85	-5.04	0.082	5'UTR;1stExon	Island
cg15306595	171673272	0.052	0.050	-4.16	-4.22	0.324	5'UTR;1stExon	Island
cg19538089	171673547	0.075	0.074	-3.65	-3.67	0.780	5'UTR;1stExon	Island
cg26391350	171673572	0.048	0.047	-4.21	-4.30	0.139	5'UTR	Island
cg16911423	171673866	0.093	0.095	-3.33	-3.30	0.545	5'UTR	Island
cg01763173	171674437	0.023	0.023	-5.46	-5.46	0.941	5'UTR	Island
cg11281641	171674855	0.049	0.047	-4.26	-4.32	0.424	5'UTR	Island
cg07420274	171676306	0.331	0.342	-0.99	-1.00	0.919	Body	N_Shore
cg01089249	171676553	0.187	0.187	-2.15	-2.14	0.953	Body	Island
cg01089319	171676809	0.172	0.175	-2.29	-2.28	0.847	Body	Island
cg14005211	171676925	0.225	0.232	-1.85	-1.81	0.701	Body	Island
cg14486905	171677602	0.298	0.305	-1.29	-1.27	0.808	Body	N_Shore
cg09144707	171678251	0.252	0.258	-1.63	-1.59	0.626	Body	N_Shore
cg02723395	171678751	0.121	0.126	-2.93	-2.90	0.813	Body	Island
cg15126544	171678954	0.030	0.028	-4.88	-5.05	0.148	Body	Island
cg04105250	171679114	0.075	0.077	-3.71	-3.70	0.972	Body	Island
cg00729049	171679402	0.146	0.136	-2.43	-2.59	0.148	Body	Island
cg15753746	171679591	0.069	0.063	-3.65	-3.81	0.123	Body	Island
cg21535772	171679906	0.138	0.141	-2.70	-2.68	0.799	Body	Island
cg08863440	171680337	0.111	0.112	-3.05	-3.11	0.704	Body	Island
cg11348701	171704223	0.876	0.885	2.81	2.89	0.328	Body	
cg14914809	171705073	0.875	0.885	2.70	2.84	0.092	Body	
cg00224929	171705463	0.931	0.929	3.74	3.75	0.918	Body	
cg03865668	171717400	0.651	0.665	0.80	0.91	0.158	3'UTR	

Abbreviation: TSS, transcription start site

UTR, untranslated region

[a] Each category of “relation to CpG island” column defines the following regions: Island, CpG island; N_Shore, 0-2kb upstream of CpG island.

Table S3. Results of probes in the *SLC6A4* region on chromosome 17

Target ID	Position (hg19)	Mean β value		Mean Adjusted M value		P value	Location with respect to *SLC6A4*	Relation to CpG Islad[a]
		PD	Control	PD	Control			
cg20592995	28524160	0.916	0.920	3.37	3.47	0.181	3'UTR	
cg24984698	28548496	0.883	0.879	3.02	2.94	0.176	Body	
cg26126367	28559497	0.964	0.964	4.75	4.77	0.793	5'UTR	N_Shelf
cg05951817	28562142	0.784	0.783	1.95	1.90	0.510	5'UTR	N_Shore
cg22584138	28562220	0.570	0.555	0.52	0.42	0.277	5'UTR	N_Shore
cg14692377	28562685	0.125	0.129	-2.89	-2.83	0.279	1stExon;5'UTR	Island
cg05016953	28562813	0.029	0.028	-5.11	-5.13	0.817	1stExon;5'UTR	Island
cg25725890	28563054	0.045	0.046	-4.45	-4.42	0.554	TSS200	Island
cg26741280	28563089	0.231	0.235	-1.79	-1.75	0.079	TSS200	Island
cg10901968	28563108	0.045	0.046	-4.44	-4.43	0.794	TSS200	Island
cg27569822	28563119	0.040	0.040	-4.59	-4.60	0.834	TSS200	Island
cg18584905	28563300	0.094	0.096	-3.40	-3.31	0.071	TSS1500	S_Shore
cg06841846	28564094	0.127	0.122	-2.77	-2.82	0.491	TSS1500	S_Shore
cg12074493	28564117	0.123	0.119	-2.87	-2.88	0.867	TSS1500	S_Shore

Abbreviation: TSS, transcription start site
UTR, untranslated region

[a] Each category of "relation to CpG island" column defines the following regions: Island, CpG island; N_Shore, 0-2kb upstream of CpG island; S_Shore, 0-2kb downstream of CpG island; N_Shelf, 2-4kb upstream of CpG island.

Table S4. Result of probes in the *SLC6A2* region on chromosome 16

Target ID	Position (hg19)	Mean β value		Mean Adjusted M value		P value	Location with respect to *SLC6A2*	Relation to CpG Islad[a]
		PD	Control	PD	Control			
cg02693870	55689324	0.425	0.437	-0.56	-0.46	0.337	TSS1500	N_Shore
cg27047406	55689534	0.214	0.222	-2.00	-1.91	0.215	TSS1500	N_Shore
cg10362591	55689865	0.088	0.088	-3.45	-3.44	0.915	TSS1500	Island
cg09746736	55690130	0.121	0.125	-2.96	-2.92	0.718	TSS1500	Island
cg16629702	55690248	0.046	0.042	-4.25	-4.42	0.024	TSS1500	Island
cg03226000	55690378	0.111	0.108	-2.94	-3.02	0.341	TSS200	Island
cg09774787	55690381	0.041	0.038	-4.43	-4.60	0.087	TSS200	Island
cg04490714	55690564	0.053	0.053	-4.19	-4.21	0.789	1stExon;5'UTR	Island
cg04874129	55690873	0.268	0.267	-1.47	-1.49	0.842	1stExon	Island
cg03860054	55691102	0.706	0.686	1.38	1.27	0.090	Body	S_Shore
cg03519481	55705229	0.924	0.928	3.57	3.65	0.355	Body	
cg05067406	55705694	0.906	0.911	3.25	3.32	0.392	Body	
cg02935718	55718768	0.933	0.934	3.80	3.83	0.629	Body	
cg04450501	55718994	0.940	0.942	4.01	4.03	0.739	Body	
cg02471751	55732070	0.955	0.956	4.42	4.44	0.739	Body	
cg04313338	55735794	0.177	0.180	-2.31	-2.25	0.296	Body	
cg27579359	55736319	0.849	0.851	2.48	2.50	0.555	3'UTR	

Abbreviation: TSS, transcription start site
UTR, untranslated region

[a] Each category of “relation to CpG island” column defines the following regions: Island, CpG island; N_Shore, 0-2kb upstream of CpG island; S_Shore, 0-2kb downstream of CpG island.

Table S5. Result of probes in the *MAOA* region on chromosome X

Target ID	Position (hg19)	Mean β value		Mean Adjusted M value		P value	Location with respect to *MAOA* [a]	Relation to CpG Islad[b]
		PD	Control	PD	Control			
cg26741576	43513914	0.128	0.124	-2.72	-2.78	0.381	TSS1500	N_Shore
cg17788031	43514189	0.497	0.500	-0.01	-0.01	0.976	TSS1500	Island
cg20121427	43514483	0.522	0.526	0.11	0.13	0.676	TSS1500	Island
cg06558952	43514718	0.233	0.227	-1.78	-1.79	0.956	TSS1500	Island
cg05443523	43515213	0.313	0.300	-0.97	-1.12	0.07364	TSS200	N_Shore
cg18138788	43515245	0.119	0.113	-2.76	-2.91	0.215	TSS200	N_Shore
cg19441691	43515349	0.105	0.105	-3.09	-3.10	0.882	TSS200	Island
cg22366618	43515377	0.500	0.504	-0.01	0.00	0.8174	TSS200	Island
cg14191108	43515457	0.566	0.564	0.39	0.38	0.85564	1stExon;5'UTR	Island
cg15014034	43515544	0.134	0.129	-2.58	-2.69	0.1446	1stExon;5'UTR	Island
cg04406445	43515575	0.510	0.512	0.05	0.06	0.856	1stExon;5'UTR	Island
cg11390960	43517029	0.850	0.847	2.68	2.61	0.699	Body	S_Shore
cg06794323	43518105	0.884	0.884	2.74	2.97	0.2379	Body	S_Shelf

Abbreviation: TSS, transcription start site
UTR, untranslated region

[a] The case-control association analysis was performed only with the female samples (PD: N = 31, Control: N = 31), as *MAOA* locates on X chromosome.

[b] Each category of “relation to CpG island” column defines the following regions: Island, CpG island; N_Shore, 0-2kb upstream of CpG island; S_Shore, 0-2kb downstream of CpG island; S_Shelf, 2-4kb downstream of CpG island.
