# Supplementary material for: Epigenome-wide association study of DNA methylation in panic disorder
Source: Clin Epigenetics. 2017 Jan 21;9:6. doi: 10.1186/s13148-016-0307-1 (PMC5270210; doi:10.1186/s13148-016-0307-1)
Supplement: Additional file 3: Table S6 and S7. — Blood-brain correlations of the significant CpG sites and meQTL sites found in the significant CpG sites. (PDF 40 kb) [file 13148_2016_307_MOESM3_ESM.pdf]

**Table S6.** Blood-brain correlations of the significant CpG sites

| Target ID | Correlation coefficients of DNA methylation between blood and brain regions | Associated brain regions |
|---|---|---|
| cg04266864 | 0.346 | EC |
| cg11029475 | 0.354 | EC |
| cg10256219 | 0.389; 0.326; 0.418 | EC; STG; CER |
| cg24247482 | 0.630; 0.571; 0.651; 0.363 | PFC; EC; STG; CER |
| cg13065121 | 0.314 | CER |
| cg26997880 | 0.318 | PFC |
| cg19464320 | 0.701; 0.714; 0.643; 0.697 | PFC; EC; STG; CER |
| cg04015759 | 0.385 | CER |
| cg15889012 | 0.377; 0.427 | PFC; EC |

Abbreviation: EC, entorhinal cortex
STG, superior temporal gyrus
CER, cerebellum
PFC, prefrontal cortex

**Table S7.** meQTL sites found in the significant CpG sites

| CHR | Position (hg19) | Target ID | Brain[a] | Blood[b] |
|---|---|---|---|---|
| 17 | 81037414 | cg25270498 | | |
| 19 | 36248877 | cg05910615 | | |
| 2 | 122407145 | cg20340149 | ● | |
| 13 | 80055594 | cg14777817 | | |
| 10 | 135088451 | cg25526061 | | |
| 17 | 27224823 | cg04266864 | | |
| 16 | 12142335 | cg10475689 | | |
| 16 | 23568708 | cg05742564 | | |
| 1 | 228604037 | cg02931001 | | |
| 13 | 28024472 | cg08209163 | | |
| 12 | 50017361 | cg10727759 | | |
| 22 | 24236284 | cg12738349 | | |
| 3 | 197409980 | cg08942682 | | |
| 6 | 170597377 | cg05228964 | | |
| 12 | 4381997 | cg08553284 | | |
| 1 | 155164676 | cg03425468 | | |
| 22 | 38202626 | cg11029475 | | |
| 16 | 2732724 | cg02205746 | | |
| 17 | 44270511 | cg10256219 | | |
| 3 | 50375496 | cg09386807 | | |
| 8 | 28243934 | cg13411962 | | |
| 4 | 4861398 | cg01959412 | | |
| 11 | 61197477 | cg03342113 | | |
| 17 | 42293627 | cg24247482 | | |
| 17 | 80189962 | cg17932802 | | |
| 1 | 204159498 | cg13065121 | | ● |
| 6 | 32055370 | cg26997880 | | |
| 12 | 121148158 | cg19464320 | | |
| 9 | 87284706 | cg13965062 | | |
| 1 | 245316477 | cg07124903 | ● | |
| 19 | 11074303 | cg08315613 | | |
| 2 | 242254519 | cg13009927 | | |
| 2 | 217559020 | cg03222971 | | |
| 1 | 206223719 | cg26795730 | | |
| 2 | 73144353 | cg15921587 | | |
| 13 | 25621328 | cg18098400 | | |
| 15 | 66993412 | cg25048202 | | |
| 2 | 27718181 | cg04015759 | | |
| 15 | 101690195 | cg24378951 | | |
| 2 | 44059266 | cg15889012 | | |

Abbreviation: CHR, chromosome

[a]meQTLs identified with brain samples by Hannon *et al.* [57].
[b]meQTLs identified with blood samples by Lemire *et al.* [58].
