# Supplementary material for: Epigenome-wide association study of DNA methylation in panic disorder
Source: Clin Epigenetics. 2017 Jan 21;9:6. doi: 10.1186/s13148-016-0307-1 (PMC5270210; doi:10.1186/s13148-016-0307-1)
Supplement: Additional file 4: Table S8. — Demographic characteristics of samples. (PDF 34 kb) [file 13148_2016_307_MOESM4_ESM.pdf]

Table S8. Demographic characteristics of samples

	PD	Control
Sample number (N)	48	48
Female/ male (N)	31/ 17	31/ 17
Average age	38.1	38.2
BMI (Average (SE))[a]	22.1 (5.0)	22.5 (3.6)
Agoraphobia rate	0.52	
Smoking status (per day)		
Non-smoking	34	
1-10	1	
10-20	4	
≥20	9	

[a] As for BMI, five missing values (one in the PD subjects and the others in the healthy control subjects) were included.
